# Metabolomics and Biomarkers for Drug Discovery

**DOI:** 10.3390/metabo8010011

**Published:** 2018-01-31

**Authors:** Pollen K. Yeung

**Affiliations:** Pharmacy and Medicine, Dalhousie University, Halifax, NS B3H 4R2, Canada; Pollen.Yeung@Dal.Ca

**Keywords:** metabolomics, biomarkers, drug discovery, personalized medicine

## Abstract

Metabolomics and biomarkers are increasingly used in drug discovery and development, and are applied to personalized medicine. Progress in these research areas has increased our understanding of disease pathology and improved therapeutic strategies for many diseases with unmet challenges. Further advances will ultimately result in the development of better drugs and breakthrough therapies, which will benefit millions of patients suffering from chronic and life-threatening diseases worldwide.

Metabolomics is an emerging science involving the study and characterization of metabolites and metabolism in biological systems using an integrated approach which generates unique chemical fingerprints for specific cellular processes [1,2]. According to the Metabolomics Society (http://metabolomicssociety.org/), metabolomics is a newly emerging field of “omics” research concerned with the comprehensive characterization of the small molecule metabolites in biological systems. It provides an overview of the metabolic status and global biochemical events associated with a cellular or biological system. As such, it can accurately and comprehensively depict both the steady-state physiological state of a cell or organism and of their dynamic responses to genetic, abiotic and biotic environmental modulation. Metabolomics focuses mainly on profiling small-molecule metabolites (metabolic profiling), and differs from genomics and proteomics, which characterize the profiles of genes and proteins, respectively. On the other hand, biomarkers are objective and quantitatively measurable indicators of biological or pathogenic processes [3], which can include small molecular entities, as well as large molecular weight proteins and genetic materials. Further, it is increasingly recognized that biomarkers and metabolomics have scientific synergies, such that metabolomics is used in many instances to identify novel biomarkers leading to the discovery of new and improved therapeutic strategies for many serious and life-threatening diseases [4,5,6].

Both metabolomics and biomarkers are increasingly used in drug discovery and development, managing disease progression and for personalized or precision medicine [2,7,8]. For examples, failures of potential disease-modifying drugs for Alzheimer’s disease (AD) may reflect the fact that participants enrolled in clinical trials are already too advanced to derive a clinical benefit. Thus, well-validated biomarkers for early detection and accurate diagnosis of AD before presentation of clinical symptoms will be crucial for therapeutic advancement. The combinatorial use of biomarkers derived from biological fluids, such as cerebrospinal fluid (CSF), with advanced molecular imaging and neuropsychological testing may eventually achieve the diagnostic sensitivity and specificity necessary to identify people in the earliest stage of the disease when drug intervention is most likely possible [9]. In another example, molecular characterization of lung cancer has changed the classification and treatment of these tumors, which is now becoming an essential component of pathologic diagnosis and oncology therapy. Advances in identifying novel biomarkers, such as epidermal growth factor receptor mutations and anaplastic lymphoma kinase translocations, have made it possible to identify subsets of patients who will benefit from targeted molecular therapies. The success of this approach has created a new paradigm for personalized therapy and led to accelerated development of new drugs for lung cancer treatment [10]. Further examples of advances include systems approaches analyzing biomarker data including inflammatory markers, neurotransmitters, lipoproteins, and hormones that have been employed successfully to identify biomarkers for aggression [11]. Significant advances have also been made using metabolomic and genomic approaches developing effective personalized diagnosis and treatment strategies for cardiovascular diseases [12,13]. Metabolic profiling has been used as a tool for prioritizing antimicrobial compounds from natural sources; in this approach, poorly expressed (cryptic) molecules are connected to their biosynthetic gene cluster which is a determining step in elucidating the biosynthetic pathway and allows downstream optimization and scaling up processes to facilitate commercial development of antibiotics [14]. 

Moreover, another advance in biomarker development is exploiting the endocannabinoid system for biomarkers for the management of sepsis. Despite advances made on many fronts in modern medicine, treatment of sepsis remains a therapeutic challenge [15]. Major challenges in improving sepsis care include developing strategies to ensure early and accurate identification and diagnosis of the disease process, improving our ability to predict outcomes and stratify patients, and the need for novel sepsis-specific treatments such as immunomodulation. Advances in biomarker development can offer promise to all three of these challenges and a solution to determining a patient’s immune status; something that is critical in guiding effective and safe immunomodulatory therapy [16]. Finally, the feasibility of exploiting ATP metabolism in the red blood cell as a sensor for energy metabolism in the body, and as surrogate biomarker for serious cardiovascular toxicities, as well as a drug target for cardiovascular protection, is increasingly being explored [17,18,19]. Advances in this research area could lead to breakthrough treatments and prevention strategies for cardiovascular and metabolic diseases which affects millions of patients world-wide.

It is clear that metabolomics and biomarkers will continue to advance drug development and personalized therapy in the future, particularly in highly demanding disease areas, such as cardiovascular and metabolic diseases, cancer, mental health and infectious disease. As the pharmaceutical industry faces increasing challenges, such as dwindling discovery pipelines, rapidly expanding research and development budgets, increasing regulatory control, significant gaps in the future drug markets, and the catastrophic consequences of ever-increasing attrition rates [5], adoption of the biomarker and metabolomics approach in drug development may provide some fresh air and help to reverse this gloomy trend. It is prudent for the industry to shift the research and development focus from speed to quality. As neatly highlighted by Woodcock in 2009, the imperative to produce high-value, innovative drugs will intensify, creating a higher performance hurdle for new therapeutics. Basic pharmaceutical and biomedical sciences will churn out candidate biomarkers with tantalizing potential to improve value, whereas methods and resources to use them effectively in drug development will evolve more slowly. The balance between these forces may well determine the success or failure of the drug development enterprise over the next decade [20].
